# The Multifaceted Dimensions of Food Choice and Nutrition

**DOI:** 10.3390/nu12020502

**Published:** 2020-02-16

**Authors:** Federico J. A. Perez-Cueto, Annemarie Olsen

**Affiliations:** Department of Food Science, University of Copenhagen, 1165 Copenhagen, Denmark; ano@food.ku.dk

The Special Issue “Food Choice and Nutrition” deals with the relationship between the food choices of different population groups or consumer segments and its impact on the nutritional status, improvement of dietary quality, food and nutrition-related behaviour, food preferences, taste education, sensory characteristics of foods and their role in consumer choice, etc. It addresses why people make certain food choices when they choose some foods instead of others, the influence of those around them on their choices, and the aspect of where foods are eaten or chosen.

This Special Issue is a comprehensive work that covers a multi-disciplinary array of studies from classic public health and nutritional epidemiology [1,2,3] to applied food technology [4,5], and covers different population groups throughout their lifespan, from pregnant women [6], children [7,8], young adults [9,10], adults [5,11,12] to older people [13].

Two reviews are included in this Special Issue. The first provides a rapid review of recent systematic reviews of food choice and nutrition, listing the most common relevant indicators, and the key message that combining interventions seems to be the most efficient way to achieve behaviour change [14]. The second provides an update on “nudging” interventions, aimed specifically at improving cardiovascular health, and highlights the fact that that traffic light-style labels, reducing portion sizes and dishware size in foodservice, and designing healthier meals are a promising methods of intervention [15].

Interventions to improve Food Security in the USA, like the Supplemental Nutrition Program for Women, Infants and Children (WIC) showed that the shorter the distance to the healthy food outlet, the higher the chance that “accessibility” is positively associated with actual fruit and vegetable intake. The paper also underscores the potential of using combined data from Geographic Information Systems and self-reported consumer data [3]. A similar approach using large population data was applied to food based dietary guidelines, with specifications of portion sizes for occasionally consumed foods e.g., those containing salt, saturated fat or those that are ultra-processed [2].

Papers from the food technology arena focus on how food reformulation can address the challenges currently faced by the meat sector [4], showing that innovation does not always mean making “new products” but can imply evaluating existing ones and improving them. Food reformulation in different categories may contribute synergically to healthier food intake at household level [5]. From a cross-disciplinary perspective, food innovation specifically in plant-based foods needs to take into account current evidence linking positive health outcomes to foods that are less processed and that have clean labels (with few or no additives) [1].

Boys are more sensitive to taste, while girls are more curious about foods [7], and therefore, any measures to improve healthier and more sustainable eating among children must focus on providing foods they like, as this will increase actual consumption and reduce plate waste. Therefore, the role of the sensory characteristics of food cannot be neglected when developing any strategies aimed at children, e.g., the promotion of more vegetable or plant-based consumption, considering that over 60% of cooked vegetables served in French school lunches are wasted [8].

Cultural beliefs and food taboos have an impact on food choices [6], an aspect often neglected in food and nutrition research. Providing culturally acceptable diets is part of the larger concept of sustainable diets and needs to be taken into account when developing the innovative foods of tomorrow. Although it is often believed that older consumers are inflexible in changing their dietary habits, such change can be achieved, e.g., starting with those older people who share a more “green” attitude [13]. In turn, changing population attitudes towards greener consumption could be a path of learning that may be extrapolated to mainstream consumers.

The provision of information in the form of labels is high in the agenda of the food and nutrition sector. Traditionally, such labelling has been consistent across many consumer groups, but tailoring to specific markets can increase the market share and consumers’ willingness to pay for a given product [16]. How such tailoring is made will require more in-depth knowledge of the local community and how it relates to other consumer segments.

The need to individualise strategies to improve nutritional status was exemplified first with the case of pregnant women, where eating behaviour was influenced by mental and physical health, body dissatisfaction, gender roles, and perceived obstacles to eating healthily [17]. Secondly, there is evidence that a reduction in portion sizes seemed more acceptable than the introduction of innovative products for French consumers [18]. Thirdly, high-performance athletes choose their foods based mainly on nutritional attributes, sensory attributes, usual eating practices (whether healthy or not), performance and on their physiological needs [10], while taste, price and hygiene were more important to truck drivers [11]. Finally, the Internet-based provision of recipes contributes to the application of healthy eating recommendations at home, but this should be in synergy with inter-generational recipes [19]. Taken together, these findings show that health promotion strategies must be better targeted and more comprehensive (including the support of mental well-being, promotion of healthier foods, improved food security, the cost of healthy foods and culturally relevant messages), rather than simple information provision on how to eat well.

This Special Issue illustrates how food choice and nutrition can be addressed from many different disciplinary perspectives and methodologies. Often, only one or a few are addressed within a single study. However, the system of food choice and how it affects nutritional status is a complex network of determinants, a complexity which is often overlooked in mono-disciplinary research. Designing the innovative, healthy, sustainable and tasty products and services of tomorrow requires a cross-disciplinary approach.
